# Built On-orbit Robotically assembled Gigatruss (BORG): A mixed assembly architecture trade study

**DOI:** 10.3389/frobt.2023.1109131

**Published:** 2023-02-22

**Authors:** Samantha Chapin, Holly Everson, William Chapin, Amy Quartaro, Erik Komendera

**Affiliations:** Field and Space Experimental Robotics (FASER) Laboratory, Virginia Polytechnic Institute and State University, Mechanical Engineering Department, Blacksburg, VA, United States

**Keywords:** ISAM, robotics, truss, structure, deployables

## Abstract

This paper explores a mixed assembly architecture trade study for a Built On-orbit Robotically assembled Gigatruss (BORG). Robotic in-space assembly (ISA) and servicing is a crucial field to expand endeavors in space. Currently, large structures in space are commonly only deployable and must be efficiently folded and packed into a launch vehicle (LV) and then deployed perfectly for operational status to be achieved. To actualize being able to build increasingly large structures in space, this scheme becomes less feasible, being constrained by LV volume and mass requirements. ISA allows the use of multiple launches to create even larger structures. The common ISA proposals consist of either strut-by-strut or multiple deployable module construction methodologies. In this paper, a mixed assembly scheme is explored and a trade study is conducted on its possible advantages with respect to many phases of a mission: 1) manufacturing, 2) stowage and transport, 3) ISA, and 4) servicing. Finally, a weighted decision matrix was created to help compare the various advantages and disadvantages of different architectural schemes.

## 1 Introduction

Driven by cramped payload fairings, complex deployment methods, and increasing launch costs, autonomous in-space assembly (ISA) serves as a space-efficient alternative, which utilizes robots to perform intricate construction tasks. With this, the next wave of satellites, space structures, and off-world habitats can be constructed.

Development of ISA and servicing techniques is ongoing. Examples include the assembly of the International Space Station (ISS) and the robotic swapping of old or failed components with new ones retrieved from a launch fairing (Azria and Belzile, 2013). Continuing efforts such as On-orbit Servicing, Assembly, and Manufacturing (OSAM) Harbaugh (2021) ^1^ and Harbaugh (2022) ^2^ missions and the Robotic Servicing of Geosynchronous Satellites (RSGS) Saplan (2022) ^3^ missions are trying to push the state of the art. To date, there have been no flown missions for large-scale robotic assembly of a truss structure. Additionally, these other missions have limited autonomous operations, demonstrating rendezvous proximity operations, but additional assembly/servicing operations are planned to be teleoperated from the ground.

The Virginia Tech Field and Space Experimental Robotics (FASER) Laboratory has identified several key research advancements required to perform robotic assembly of large-scale structures in space. Further development and investigation are required to achieve an ideal modular design for scalable, repeated space structures. Advanced local metrology methods must be developed to facilitate the assembly of large structures, where employing a global metrology system would be impractical. Collaborative assembly between heterogeneous robots must be achieved to allow for the optimization of assembly steps depending on the skill set of the available robots. Structural damage detection is required to either mitigate errors during assembly or identify them post-assembly and facilitate servicing. Finally, increasing confidence in autonomy is crucial to allow end-to-end autonomous operations.

This paper describes the conducted trade study to determine if a mixed assembly architecture could compare to the more commonly considered strut-by-strut or full deployable truss assembly schemes. The strut-by-strut scheme involves using single struts and assembling them to create 3D truss structures. The fully deployable truss scheme involves designing the truss to be able to fold up for launching and be deployed in-space to its full dimensions. Finally, the mixed assembly scheme evaluated in this paper involves having some deployable modules connected at corners to create a checkerboard pattern, where the mission edges and planes are completed with close-out-struts and close-out-squares, respectively. The FASER Laboratory plans to conduct a scaled-down assembly demonstration, but first, a trade study was required for this mixed assembly scheme. This paper will investigate the manufacturing, stowage and transport, ISA, and servicing comparisons between the strut-by-strut, full deployable, and mixed assembly schemes.

## 2 Materials and methods

### 2.1 In-space assembly and servicing background

#### 2.1.1 Robotics

ISA at large scale requires much more effort, material, and time than human assembly. Hence, robotic capabilities are required. Space robotics has a long history, which encompasses the sum total of exploration to every body in our Solar System except for the Moon landings. Robotic capabilities in space range from ground-based rovers and landers, interplanetary probes, satellites, human aids such as Robonaut (Deiss, 2022)^4^, and dextrous manipulators such as Canadarm Canadian Space Agency (2019) ^5^. For the purposes of ISA, dexterous manipulation, inspection, and human aid categories are the most significant.

Thus, by far, ISA activities have been demonstrated only with space stations such as the ISS and Mir and with the Space Shuttle. No ISA activities have been completed or attempted outside of low Earth orbit (LEO). As the MIR and shuttle programs have been discontinued, the ISS remains the only host of contemporary ISA demonstrations (though future missions are slated for launch and demonstration). The ISS has onboard capabilities for dexterous robotic manipulation in the Mobile Servicing System (MSS), which consists of the Space Station Remote Manipulator System (SSRMS), also known as Canadarm2, the Mobile Remote Servicer Base System (MBS), and the Special Purpose Dexterous Manipulator (SPDM), better known as Dextre. The ISS is also host to a number of smaller arms including the Japanese Experiment Remote Manipulator System (JEMRMS) and the European Robotic Arm (ERA) Currie and Peacock (2002).

Robots used to interact with and aid astronauts have been developed and tested in a variety of form factors. Free fliers such as AERCam sprint and Astrobee have flown and interacted with astronauts in both pressurized environments and in vacuum (Nanjangud et al., 2018). Humanoid robots meant for use in close proximity with astronauts and with tools and interfaces designed for human manipulation have also been flown, including Robonauts 1 and 2. The successor of the Robonaut program, Valkyrie, is currently under development at NASA’s Johnson Space Center (Kisliuk, 2017) ^6^.

The ongoing research into ISA and servicing can be divided into component assembly, component manufacture, and structure modification or repair. Research into the component assembly area has included the Commercial Infrastructure for Robotic Assembly and Services (CIRAS) project (Wong et al., 2018), which was led by Northrup Grumman. The project aimed to develop the capabilities for assembling square bay robotic trusses on-orbit. To this end, Northrup Grumman collaborated with NASA Langley Research Center (LaRC) to use the existing Tension-Actuated Long-reach In-Space Manipulator (TALISMAN) (Doggett et al., 2015) and develop the NASA Intelligent Jigging and Assembly Robot (NINJAR), which were used in the deployment of a square bay truss structure with the subsequent attachment of solar array components, and the assembly strut-by-strut of a two square bay truss, respectively. Research into the component manufacturing area has centered on additive manufacturing techniques. Made in Space has demonstrated 3D printing on the ISS as early as 2014 (Boyd et al., 2017). Made in Space is also participating in On-orbit Servicing, Assembly, and Manufacturing (OSAM) 2 with the Archinaut project, which aims to enable large-scale orbital additive manufacturing. Made in Space demonstrated the capability in a thermal vacuum chamber in 2017 located at NASA’s Ames Research Center. Research into the structure modification or repair area includes OSAM-1, which aims to extend the lives of satellites that were not designed with robotic servicing in mind (Coll et al., 2020). DARPA’s Robotic Servicing of Geosynchronous Satellites (RSGS) (Saplan, 2022) ^7^ consists of similar mission objectives and is being developed in parallel.

For the purposes of this paper, all described assembly methods fall under the umbrella of component assembly, so the most desired capabilities are those offered by Long Reach Manipulators (LRMs) and Dexterous Manipulators (DMs). All required steps described herein fall under the capabilities of the LRMs and DMs either installed on the ISS or in research and development on the ground. Therefore, a combination of a LRM and a DM serving as the LRM’s end effector (EE) was selected on the basis of these described assembly procedures. This combination can be seen in the ISS’s Canadarm2 and Dextre, or NASA LaRC’s TALISMAN and Stewart platform robotic pairs. These combinations combine the mobility of the LRM and the precision for robotic operations of the DM. Other robotic methods such as free-flyers and truss-walkers could be used for the ISA concepts discussed here. However, the LRM and DM tandem team will be utilized in all further operational discussions.

#### 2.1.2 Structures

In 2021, NASA LaRC published a survey of their research into large space structures (Dorsey et al., 2021), which is summarized as follows. In the 1960s, two concepts were developed for a large space reflector and a communication satellite. In the 1980s, LaRC conducted testing of a 5 m cubic truss, with 2 in diameter struts, for the main structure of the proposed Space Station Freedom (SSF), using an erectable node and a joint system and testing simulated ISA in a neutral buoyancy tank (Watson et al., 1988) ^8^. Then, the SSF truss was adapted to a scaled-down 1-in cross-sectional diameter version and changed from a cubic framework to a tetrahedral structure to test a precision segmented reflector (PSR) concept. This involved assembling a 14 m test structure with a team of two astronauts in a neutral buoyancy tank (Bush et al., 1991) (Pawlik et al., 1989). In the early 2000s, NASA LaRC also investigated assembling truss structures using robots in their Automated Structures Assembly Laboratory (ASAL), where they assembled an example telescope backplane tetrahedral truss structure from 102 2-m long struts (Doggett, 2002). Newer telescope structures are in development called Tri-Trusses, which are a concept for a deployable truss that could be assembled to create the backplane of a large space telescope on the order of 20 m in diameter (William et al., 2019). In 1985, NASA conducted the Assembly Concept for Construction of Erectable Space Structure (ACCESS) experiment during an Extravehicular Activity (EVA) outside the shuttle where two astronauts assembled a 13.7 m long structure (Heard et al., 1986). In 2017, as a part of the previously mentioned CIRAS project, NASA LaRC assembled a 32-in cubic truss using a team of robots. This was a strut-by-strut assembly where struts were grabbed by the Strut Attachment, Manipulation, and Utility Robotic Aide (SAMURAI) at the end of a LRM, and then handed off to the NINJAR, which precisely positioned them until a full truss bay was assembled and then lifted (Wong et al., 2018). Additional truss bays could then be constructed below to create any tower of any consisting desired bays. Overall, there are many other examples of structures in space, but this shows how many examples can be found for either assembling a truss strut-by-strut or assembling deployed units either robotically or with astronauts. The idea of having a mixed assembly scheme, as has been described in this paper, could not be found previously published though it is obvious that mixed methods of assembling and deploying structures are heavily used in space exploration. For example, the ISS was created with a mixture of launching prefabricated modules, docking them together to assemble, and deploying large truss units such as those for the solar arrays to create the final station structure and desired functionality.

### 2.2 Built On-orbit Robotically assembled Gigatruss Concept

The Built On-orbit Robotically assembled Gigatruss (BORG) concept is focused on testing autonomous robotic operations for assembling a hybrid truss structure consisting of deployed and strut-assembled elements. This mixed assembly approach combines the quick assembly time of a deployable structure with the small launch volume of a piece-by-piece assembly system.

The assembly uses a checkerboard scheme with deployable units connecting at corners and completing the structure with close-out elements to reduce strut redundancy. This approach will be tested at FASER by creating a 3 × 3 × 3 unit truss structure. Each deployable structure will be a cube 3 m on a side, resulting in a cubic truss 9 m on a side. The number of modules and dimensions used in this demonstration were chosen arbitrarily, as the design is scalable to any number of modules and dimensions.

For this construction, the BORG cube will be assembled by a team of heterogeneous robots. The DM unit will be used for precision manipulation and support of the structure throughout the assembly processes. The DM will be outfitted as an EE on the LRM, which provides gross movement capability. With a repeated assembly scheme, additional layers in every direction can be added to construct a larger free-standing BORG truss with no additional complexity.

### 2.3 BORG truss structure

**FIGURE 1:**
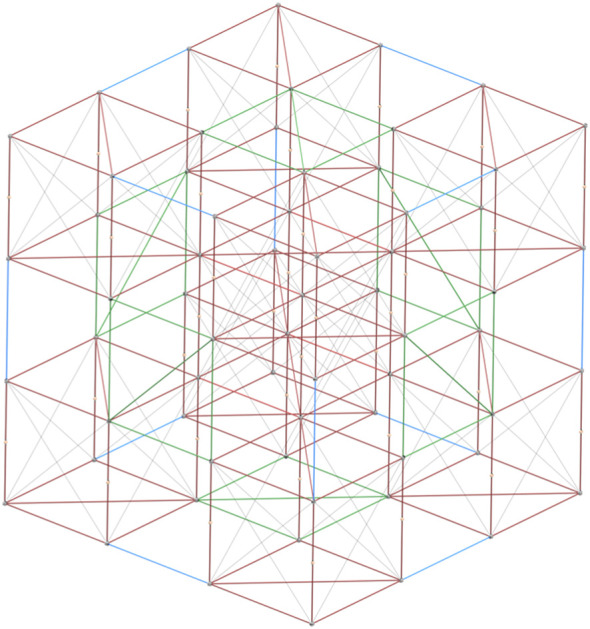
An example of the BORG truss structure with 3 × 3 × 3 units. Deployable units are shown in red with close-out-squares in green and close-out-struts in blue.

**FIGURE 2:**
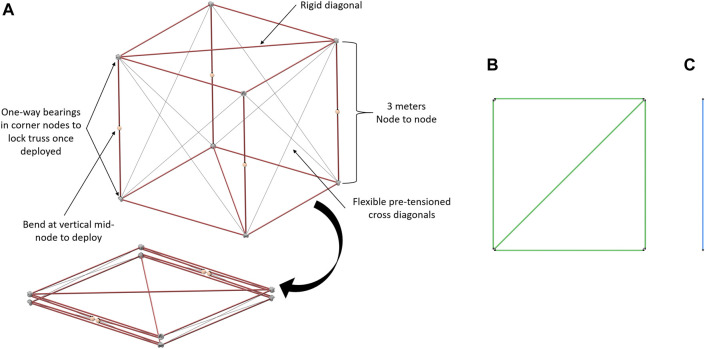
Three types of BORG truss modules: **(A)** deployable truss: uses one-way bearing in corner nodes to lock truss once deployed, bends at the vertical mid-node to deploy, rigid diagonals, and horizontal struts, flexible pre-tensioned cross diagonals, **(B)** close-out-square: corner nodes have features to guide in close-out struts and squares and then lock them into place with a spring pin, and **(C)** close-out-strut: a strut with two ends that insert into the corner nodes.

The 3 × 3 × 3 example BORG truss as seen in Figure 1 consists of three unique modules: nine deployable trusses, six close-out squares, and twelve close-out struts. These three units are depicted in Figure 2. This deployable truss framework is a 1-unit square bay truss with rigid horizontal struts, vertical struts that hinge at the mid-node to facilitate deployment, and flexible pretensioned cable diagonals to allow applied compression. The corner nodes have unique geometries for assembly, deployment locking mechanisms, and can be pre-integrated with 3-axis electrical connections to provide continual electrical through-lines throughout the structure. Deployable diagonals will use cabling pretensioned to 15 percent of maximum potential stress *via* turnbuckles, adding to the overall stiffness of the assembled structure. The other two close-out structures contain either a single strut or a rigid square with a diagonal element for connecting and stabilizing multiple deployable bays. With this configuration, the deployable structure can have a tighter packing volume than a fully rigid structure while utilizing far fewer assembly components when compared to a solely strut-by-strut assembly.

### 2.4 Mixed Assembly Concept of Operations (CONOPS)

Following are the steps required to assemble each layer of an example BORG truss using a LRM equipped with a DM EE. The checkerboard pattern of connecting deployable units alternates with each additional layer. So as seen in each new “slice” of the cubic structure it alternates which location has the deployable structure. This alternating deployable pattern makes up the interior of the truss structure where the exterior closes the gaps in the structure with close-out-square connecting deployable units on the exterior faces and close-out-struts connecting the deployables on exterior edges. The smallest scale this methodology is possible at is 3 × 3 × 3 units as it provides the opportunity for close-out structures. To utilize only the three truss modules shown previously, the BORG structure must also have an odd number of bays to allow for closeout structures. With this, it is theoretically possible to extend this structure *ad infinitum,* if desired.

**FIGURE 3:**
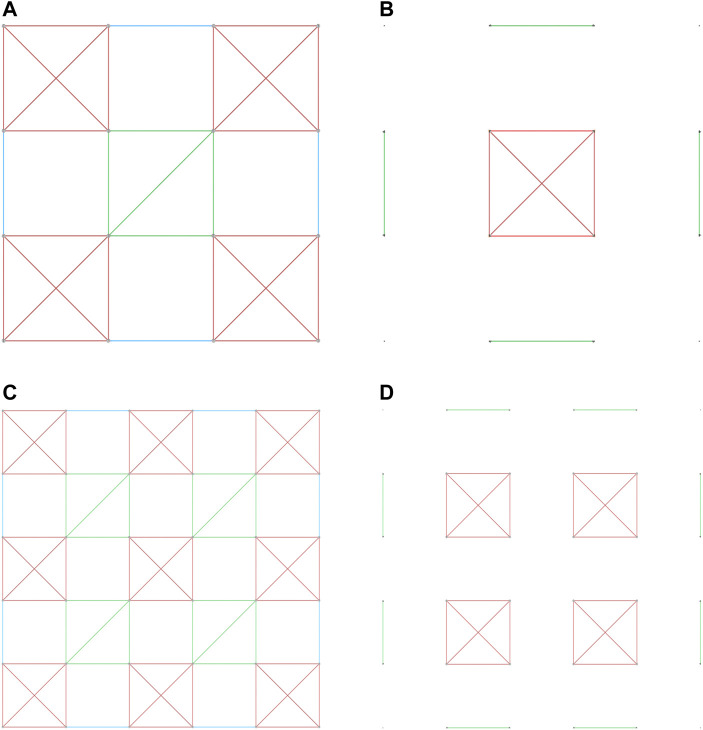
For a 3 × 3 × 3 [**(A)** layer 1 and 3; **(B)** layer 2]. For a 5 × 5 × 5 [**(C)** layer 1, 3 (layer 3 replaces the close-out-squares with deployable cubes), and 5 **(D)** layer 2 and 4].

A top–down view of each layer is shown in Figure 3, where again the deployable module is shown in red, the close-out-square is shown in green, and the close-out-strut is shown in blue. Figures 3A, B show the assembly scheme for the 3 × 3 × 3 unit, whereas Figures 3C, D show it at the 5 × 5 × 5 size to show how the scheme scales.

**FIGURE 4:**
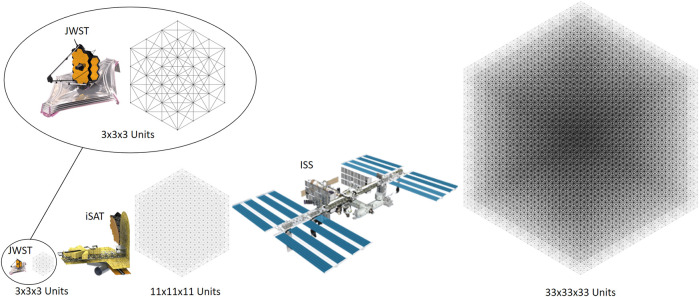
Comparison of sizes of BORG structures analyzed with detailed view of JWST and 3 × 3 × 3. JWST NASA (2022c), iSAT NASA (2022b), and ISS NASA (2022a). Image Credit: NASA.

An example conops breaking down the 3 × 3 × 3 structure shown in Figure 3 is as follows:

Layer 1—3 × 3 × 3

Following are the three steps of the Layer 1 assembly process for creating Layer 1 of Figure 3:

1. For one square: DM brings close-out-square to the assembly area and places it
2. For four deployable units: LRM with DM EE retrieves stowed deployable truss and deploys each truss unit before placing them into their corresponding four corner locations, attached to the center close-out-square by one corner
3. For four struts: Use the DM to hold adjacent truss units in place while the close-out-strut is placed

Layer 2—3 × 3 × 3

Following are the three steps of the Layer 2 assembly process for creating Layer 2 of Figure 3:

1. For one deployable unit: LRM with DM EE retrieves stowed deployable truss, deploys the truss, and then, places it into position
2. For four struts: Use the DM to hold adjacent truss units in place while close-out-strut is placed
3. For four squares: DM brings close-out-square to the assembly area and places it and repeats with the remaining three close-out-squares

Layer 3 - 3 × 3 × 3

Following are the three steps of the Layer 3 assembly process for creating Layer 3 of Figure 3:

1. For four deployable units: LRM with DM EE retrieves each stowed deployable truss one at a time and deploys them before placing them into the corner positions
2. For four struts: Use the DM to hold adjacent truss units in place while close-out-strut is placed
3. For one square: DM brings close-out-square to the assembly area and places it

## 3 Results

When comparing the different methods to either deploy, construct, or complete a mixture of the two in order to end up with a large structure in space, four different phases of the mission were considered: 1) manufacturing, 2) stowage and transport, 3) ISA, and 4) servicing. For each stage, the strut-by-strut, full deployable, and mixed assembly schemes to create a BORG truss were examined. Another assembly scheme of assembling all deployable modules was not analyzed due to the large number of overlapping members it would generate, which would result in it performing extremely poorly in mass and volume-dependent situations compared to the other assembly methods. In addition, three different sizes of end trusses, each seen in Figure 4, are analyzed: a 3 × 3 × 3 unit BORG, an 11 × 11 × 11 BORG, and a 33 × 33 × 33 unit BORG. For all of these, a base unit of a 3-m cubic truss was used for consistency but as the structure gets larger, the argument for having a larger base unit could be made. The 3 × 3 × 3 was chosen since its final dimensions of 9 m cube is on par with communication satellites and state-of-the-art space telescopes such as the James Webb Space Telescope (JWST) NASA (2022b) ^9^. In addition, the 3 × 3 × 3 case represents the smallest configuration that allows the mixed assembly scheme to work due to the checkerboard design. The next size of 11 × 11 × 11 was chosen because the end dimension of 33 m per side is approximately the size of the next generation of large space telescopes proposed in the in-Space Telescope (iSAT) study (Mukherjee, 2019) ^10^. The proposed telescope’s aperture of 20 m or greater could provide the next great leap in scientific discovery, and thus would require a large support structure. Finally, 33 × 33 × 33 units were selected with a final dimension of 99 m per side because it is analogous to creating a structure on the scale of the ISS (Garcia, 2022) ^12^.

### 3.1 Manufacturing

To start comparing the architectures, we first looked at the ground manufacturing, assembly, integration, and testing that would be required to manufacture all of the modules required. When looking at how many components need to be made for each assembly or deployment scheme, the number of steps for each type of component was calculated based on the number of manufacturing and assembly tasks. Then, this number was multiplied by the number of components that were included for each scheme. For the full deployables, the number of struts was multiplied by 6 compared to a strut-by-strut approach because for simplicity, we assumed a compression factor of 6 for a full deployable using telescoping rods made of six components to achieve each 3 m strut. The results of this are shown in Table 1.

**TABLE 1:** Calculating the number of manufacturing steps for each assembly or deployable technique for all three final structure sizes. The cells highlighted in green show the architecture for each size structure with the fewest manufacturing steps required.

Type of module	Calculating number of manufacturing steps				Number of modules required per 3 × 3 × 3 architecture			Number of modules required per 11 × 11 × 11 architecture			Number of modules required per 33 × 33 × 33 architecture		
	*Make* *strut*	*Make* *node*	*Assembly* *steps*	*Total manufacturing* *steps*	*Strut-by-strut*	*Full* *deployable*	*Mixed* *assembly*	*Strut-by-strut*	*Full* *deployable*	*Mixed* *assembly*	*Strut-by-strut*	*Full* *deployable*	*Mixed* *assembly*
*Strut*	1	2	2	5	252	1512	12	9108	54648	60	225522	1353132	192
*Close-out-square*	5	4	8	17	0	0	6	0	0	150	0	0	1536
*Deployable*	26	12	37	75	0	0	9	0	0	341	0	0	9009
Manufacturing steps for architecture					1260	7560	837	45540	273240	28425	1127610	6765660	702747

Table 1 shows that for all BORG structure scales, the mixed assembly approach minimizes the manufacturing steps. The strut-by-strut approach is the next efficient, and then, the full deployable scheme is the worst.

When comparing methods, it is important to minimize manufacturing tasks in order to speed up the manufacturing time, reduce the time for checking out all the components, and reduce the number of components that can fail.

#### 3.1.1 Pre-integration of advanced capabilities during manufacturing

When thinking of manufacturing, it is also important to think of other capabilities that can be built into the structure so that those capabilities do not have to be added later once on-orbit. A few examples of such capabilities include electronics, cabling, fluid pathways, and communication lines. With a strut-by-strut approach, unless each interface has all required pass through, which would be difficult and make them more complex, heavy, costly, and prone to failure, and then, once the structure is assembled, any additional capabilities have to be added to the structure in orbit. A fully deployable unit is already very complex to design to fold up with the maximum efficiency if it needs to fit inside an available launch vehicle, leaving very little room for additional capabilities. The mixed assembly could potentially allow the ability to add more complexity into each deployable unit and then have to only add additional infrastructure in sparser areas to connect the checker-boarded deployables.

It can be posited that the mixed assembly method is a happy medium to allow the pre-integration of some components into the deployables, which localizes the risk to smaller units where failure is not as impactful while still having less manufacturing being required than the most flexible strut-by-strut approach.

### 3.2 Stowage and transport

The ability to successfully deliver structures and materials into space is paramount in the discussion of ISA. This is due to the fact that launch vehicles have constrained sizes that limit the volume available. To fit into these constrained spaces, most spacecraft launched to date have employed complex deployment methods if their final deployment volume was larger than the allotted size. This, although efficient, has its challenges, especially when approaching the next generation of large space structures. To highlight this, this section and the next will showcase that when scaling structures up, fully deployable structures hit a limit to how large they can be. As these large truss structures can be tailored by many factors, namely, the size of each individual bay and the number of bays present, a few assumptions for running a launch analysis had to be defined. As previously mentioned, the 3 × 3 × 3, 11 × 11 × 11, and 33 × 33 × 33 number of unit cases will be analyzed each with an individual bay size of 3 × 3 × 3 m. These will be true for the cases analyzed in both this section and Section 3.3.

The selection of a 3 m truss was constrained by the diameter of the launch vehicle fairings. The most common rockets currently in operation have a diameter of approximately 5 m with an interior payload envelope diameter of 4.572–4.6 m. Thus, in order to have the most launch vehicles accessible for choice, the individual deployable unit had to fit inside this cross-section. The largest square that could fit in a circle of 4.572 m diameter had an edge length of 3.233 m. For the ease of numbers and to account for any protruding nodal geometry, an edge length of 3 m was selected for all future operations. This also aligns with the current box truss architecture used in proposed ISA space structures as those usually sit between 2 and 5 m (Dorsey et al., 2021). With rocket classes of 8.4 and 10 m diameter payload fairings on the horizon, there is the opportunity for larger bays, however, at a much steeper cost.

#### 3.2.1 Packing efficiency

**FIGURE 5:**
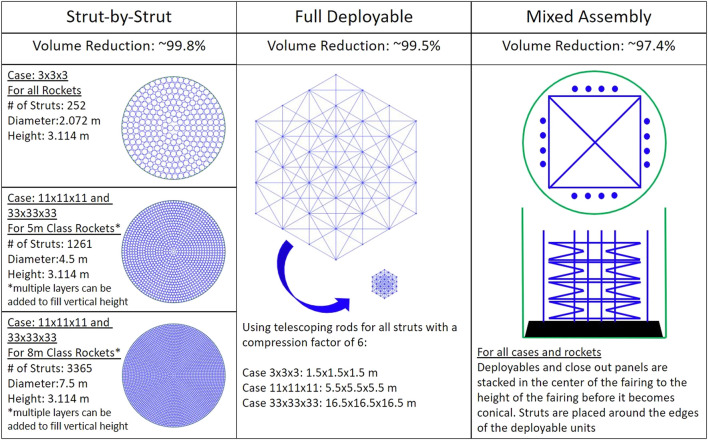
Packing style for varying architectures.

Of the most critical factors in comparing the feasibility of the following ISA schemes, packing efficiency stands paramount. This is due to the fact that the cost to launch is exorbitantly expensive, so every effort must be made to fit into the smallest rocket and the fewest number of launches to be practical. Of the three assembly methods discussed, the strut-by-strut assembly will always have the highest packing efficiency as individual struts can be packed very tightly. For most space utilization, Figure 5 shows these struts being stacked vertically in radial rings. For the 3 × 3 × 3 case, all the struts required, 252, can fit in a diameter of 2.072 m and thus can fit in any rocket payload fairing. For the 11 × 11 × 11 and 33 × 33 × 33 cases, there are two packing diameters for the two main classes of rockets. These cylinders of stowed tubing can then be stacked on top of each other if the height of the payload fairing allows.

For the fully deployable case, there are many ways to deploy a structure. Two common methods that are often used are components that simply fold up as seen in the deployable case previously mentioned and telescoping rods. There has been a long history of trying to include complex deployment mechanisms into designs in an effort to save space. Although they can often compact themselves very tightly, there will always be a limit to the size of structures that can fit in a single fairing as there is a lower limit to how much the dimensions can be compressed. This is increasingly problematic with the increase in the size of the final structure because despite the very high packing efficiency that can be achieved, it will quickly surpass the diameter of the payload fairing.

With this mixed assembly model, as shown in Figure 5, the deployables are stacked upon one another with the close-out-squares on top of that then surrounded radially by the close-out-struts stacked in the same manner as the strut-by-strut case. This pattern allows for the units to simply be stacked as tall as possible in the cylindrical height of the payload fairing.

#### 3.2.2 Launch vehicle analysis: Number of sized vehicles

As previously mentioned, when selecting launch vehicles (LVs), there exist three main classes of rockets; the 5 m, the 8.4 m, and the 10 m payload fairing sizes. Of these, the 5 m fairing class has the most options and has had the largest flight history. For this class, the following rockets were selected: Falcon nine from SpaceX (2021) ^12^, Ariane 5 from Ariane Space (2016) ^13^, Delta IV from United Launch Alliance (2013) ^14^, and Altas V also from United Launch Alliance (2010) ^15^. Many of these launch vehicles have a standard and long payload fairing, so both of those were taken into account in this study. For the 8.4 and 10 m cases, the only rocket that falls in this category is the Space Launch System (SLS) from NASA (2018) ^16^, specifically SLS Block 1a Cargo and SLS Block 1 b Cargo respectively. Starship from SpaceX (2020) ^17^ was also a valid option; however, preliminary launch costs are being stated at $10 M Berger (2022) ^18^, which is a massive outlier and so will not have its pricing compared to the other contenders. For this application, the most crucial factor limiting launch is the fairing size, but mass limit to low Earth orbit (LEO) and the cost also play key roles in the success of the launch. In Table 2, these vehicle parameters are compared. The green cells represent the lowest total cost for each individual architecture, not including the Starship cost factor as previously mentioned. The next section of this table breaks down the number of launches needed by each rocket to fully deliver three differing architectures at three size categories to space.

**TABLE 2:** Launch vehicle feature comparison with data from SpaceX (2021), Ariane Space (2016), United Launch Alliance (2013), United Launch Alliance (2010), SpaceX (2020), Berger (2022), and NASA (2018).

			Falcon 9		Ariane 5		Delta IV		Atlas V		Starship	SLS Block1a	SLS Block 1b	
						Standard	Long	Standard	Long	Standard			Long	Medium	Long	Standard	Long
Rocket properties	Total height (m)		11	16.5	12.882	15.589	11.447	15.992	12.927	16.485	22	16.52	16.52	24.83
	Internal diameter (m)		4.6		4.572		4.572		4.572		8	4.6	7.5	
	LEO mass limit (kg)		22,800		20,000		22,800		17,720		100,000+	95,000	63,500	
	Approximate cost/launch		$67 M		$156 M		$62 M		$153 M		$10 M	$2 B	$4.1 B	
3 × 3 × 3	Strut-by-strut	# of launches	1	1	1	1	1	1	1	1	1	1	1	1
		Total cost	$67 M	$67 M	$156 M	$156 M	$62 M	$62 M	$153 M	$153 M	$10 M	$2 B	$4.1 B	$4.1 B
	Deployable	# of launches	1	1	1	1	1	1	1	1	1	1	1	1
		Total cost	$67 M	$67 M	$156 M	$156 M	$62 M	$62 M	$153 M	$153 M	$10 M	$2 B	$4.1 B	$4.1 B
	Mixed assembly	# of launches	2	1	2	1	2	1	2	1	2	1	1	1
		Total cost	$134 M	$67 M	$312 M	$156 M	$124 M	$62 M	$306 M	$153 M	$20 M	$2 B	$4.1 B	$4.1 B
11 × 11 × 11	Strut-by-strut	# of launches	4	3	4	3	4	3	4	3	2	4	1	1
		Total cost	$268 M	$201 M	$624 M	$468 M	$248 M	$186 M	$612 M	$459 M	$20 M	$8 B	$4.1 B	$4.1 B
	Deployable	# of launches	X	X	X	X	X	X	X	X	1	X	X	X
		Total cost	X	X	X	X	X	X	X	X	$20 M	X	X	X
	Mixed assembly	# of launches	17	10	17	11	17	10	15	10	14	9	9	6
		Total cost	$1.14 B	$670 M	$2.65 B	$1.72 B	$1.05 B	$620 M	$2.3 B	$1.53 B	$140 M	$18.B	$36.9 B	$24.6 B
33 × 33 × 33	Strut-by-strut	# of launches	90	60	90	60	90	60	90	60	34	90	23	17
		Total cost	$6.03 B	$4.02 B	$14.04 B	$9.36 B	$5.58 B	$3.72 B	$13.77 B	$9.18 B	$340 M	$180 B	$94.3 B	$69.7 B
	Deployable	# of launches	X	X	X	X	X	X	X	X	X	X	X	X
		Total cost	X	X	X	X	X	X	X	X	X	X	X	X
	Mixed assembly	# of launches	392	216	383	262	391	233	344	216	329	210	210	128
		Total cost	$26.26 B	$14.47 B	$59.75 B	$40.87 B	$24.24 B	$14.44 B	$52.63 B	$33.05 B	$3.29 B	$420 B	$861 B	$524.8 B

Through these calculations, it can be seen that each of the three assembly styles for the 3 × 3 × 3 can be put into at least one of the rocket fairings. Some of the short length fairings would require two launches but this can simply be sidestepped by employing the longer fairing. In this 3 × 3 × 3, all three architectures would reach space with the minimum cost of $62 M aboard a single Delta IV. For the next size category of 11 × 11 × 11, the compacted deployable dimensions exceed all payload fairings apart from the Starship option. For the strut-by-strut assembly and mixed assembly methods, they can be delivered in 3 Delta IV launches for $186 M and in 10 Delta IV launches for $620 M, respectively. Unsurprising the 33 × 33 × 33 case had no hope of fitting into any current payload fairing. At this point, though, the other methods can be launched in the available rockets, the sheer number of launches needed, and the resulting costs mean that as the size approached the 33 × 33 × 33 bay option, the feasibility diminishes. The strut-by-strut option can be completed in 60 launches aboard the Delta IV rocket for $3.72 B. On the other hand, the mixed assembly scheme would take 233 Delta IV launches totaling $14.4 B.

The option of using SLS is a very tempting one. If this launch vehicle is chosen, the required number of launches is reduced by a factor of 2 in some cases and up to a factor of 3 reductions in others. However, the price per launch is $4.1 B, and the cost for the entire mission exceeds that of numerous smaller-class rocket launches. This is one of the biggest benefits of an assembly method such as strut-by-strut or the mixed model as it is able to utilize these much smaller rockets saving significant amount of money.

### 3.3 In-space assembly

For the purposes of this paper, the exact operation of ISA for each architecture is generalized to be able to compare them at a higher level. Throughout this analysis, a single robotic assembly agent is utilized for the entire assembly of the strut-by-strut or mixed assembly architectures or to deploy the fully deployable. An added aspect more crucial to the ISA architectures (*vs.* the full deployable) is robot motion planning to avoid collision, and compliance control for secure connections between modules. In addition, concepts such as what modality of assembly including teleoperation-based assembly, shared control, or autonomous assembly are simplified for this paper, and public data on general space robotic movement speed and ground robotic assembly speed have been used across the architectures to estimate assembly times agnostic the method of control.

#### 3.3.1 Scalability

When analyzing the scope of the structures proposed for ISA, trusses are repeatable architectures that can be constructed out to any desired distance. Here, the comparative scalability of the strut assembly, single deployable, and mixed assembly style trusses will be evaluated. The math to generate the number of elements needed for the architecture is as follows:

Strut-by-strut assembly scalabilty:

For x, y, and z representing the number of cells along each axis:

$$\begin{array}{l} \text{Straight Elements}=3xyz+2\left(xy+yz+xz\right)+x+y+z, \end{array}$$ (1)

$$\begin{array}{l} \text{Diagonal Elements}=3xyz+xy+xz+yz, \end{array}$$ (2)

$$\begin{array}{l} \text{Total Nodes}=\left(x+1\right)\left(y+1\right)\left(z+1\right), \end{array}$$ (3)

$$\begin{array}{l} \text{Face Nodes}=2\left(\left(x-1\right)\left(y-1\right)+\left(y-1\right)\left(z-1\right)+\left(x-1\right)\left(z-1\right)\right), \end{array}$$ (4)

$$\begin{array}{l} \text{Edge Nodes}=4\left(x+y+z-3\right), \end{array}$$ (5)

$$\begin{array}{l} \text{Corner Nodes}=8, \end{array}$$ (6)

$$\begin{array}{ll} \text{Interior Nodes} & =\text{Total Nodes}-\text{Face Nodes}-\text{Edge Nodes}\\ & \;-\text{Corner Nodes}. \end{array}$$ (7)

Fully deployable scalabilty:

The current fully deployable architecture poses that each individual strut will have a deployable component to reduce its size. If each strut only contains a single deployment step, then the number of deployments is equal to the number of struts. Therefore, equations and for the strut-by-strut assembly case hold true for this deployable case as well. If the number of deployment steps per strut were increased, these equations can simply be multiplied by a scaling factor.

Mixed assembly scalabilty:

For n as the number of cells along an edge:

$$\begin{array}{l} f=\frac{n-1}{2}, \end{array}$$ (8)

$$\begin{array}{l} \text{Number of Squares}=6*f^{2}, \end{array}$$ (9)

$$\begin{array}{l} \text{Number of Struts}=6*\left(n-1\right), \end{array}$$ (10)

$$\begin{array}{l} \text{Number of Deployables}=2f^{3}-3f^{2}+3f-1. \end{array}$$ (11)

**FIGURE 6:**
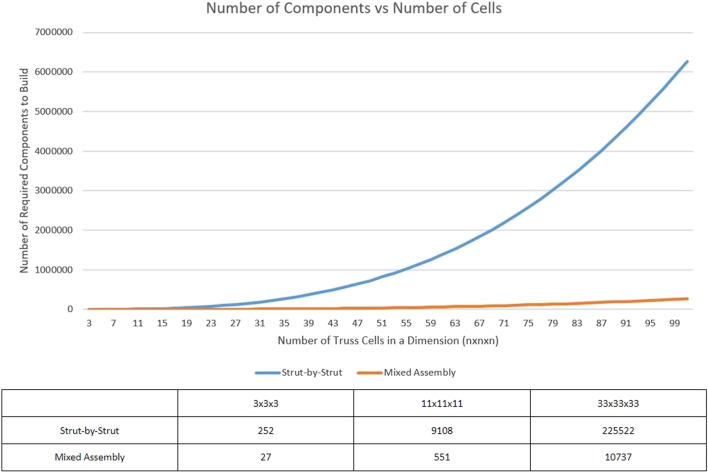
Graph comparing the number of components required to construct a truss of size nxnxn.

When comparing these formulations, the following dimension to component conclusion can be seen charted in Figure 6. Now, based on the previously shown equations, both the mixed assembly and strut-by-strut assembly models are both cubic growth functions. However, as is apparent based on the diverging lines, strut-by-strut assembly experiences a much larger growth in the number of components needed to construct the truss when compared to a mixed assembly truss of the same dimensions.

#### 3.3.2 Time to completion

In comparison to an Earth-based assembly scheme, space assembly is notoriously slow. This is further compounded in the case of robotic space assembly. This is due to many factors ranging from data collection, state estimation, motion planning, and low-velocity trajectories. However, robots are able to operate a non-stop assembly schedule that human beings would never be able to work at, especially in a highly dangerous environment such as space. The following calculations were all made under the assumption that a singular LRM and DM pair was performing all the necessary operations. The completion time would be greatly reduced if more robotic agents were contributing to the assembly space. This team of robots could help to perform tasks in tandem and work parallel operations to greatly optimize the time spent. Due to the large number of component-level tasks, the strut-by-strut and the mixed assembly schemes would benefit greatly from a team like this.

Due to these large time steps per task, the number of tasks greatly impacts the time to complete the structure. As shown in the previous section, the strut-by-strut assembly requires many more components in comparison to the mixed assembly so it is of no surprise that it will take more time to construct. But the individual tasks in the mixed assembly scheme on average take longer. For the full individual strut insertion task, a time value of 10 min was selected. This value is based on previous testing conducted by NASA Langley in the NINJAR truss assembly times (Wong et al., 2018). In these trials, the strut assembly time was roughly 13.8 min per strut. In the case of close-out-squares, a 15 min time was allotted as it has to ensure four connection points instead of the two needed for strut assembly. Finally, for the deployable task, as the structure needs to both be deployed and locked out and then placed in the full assembly, a time of 25 min was estimated.

For the time calculation of the deployable structure, the values were based on both the operational speed of Canadarm2 and the deployment of the S6 truss on the ISS. For Canadarm2, the loaded velocity during ground control was 2 cm/s Canadian Space Agency (2019) ^19^ and the deployment during the first half of the procedure extended at roughly 6 cm/s (Pearlman, 2009) ^20^. Both these speeds are extremely fast for space robotic deployment especially when autonomy is included. Although the architecture style of deployment is extremely different from a cubic truss deployment, the JWST took approximately 5 days to deploy its Sun shield although most of this time was spent monitoring and adjusting (Bartels, 2021) ^21^. Thus, a speed of 1 cm/s was chosen which is still very quick, and although this may be an option for deployment velocity, the system would pause at each time step to update positional data, the motion plan, and then carry out the following time step.

**FIGURE 7:**
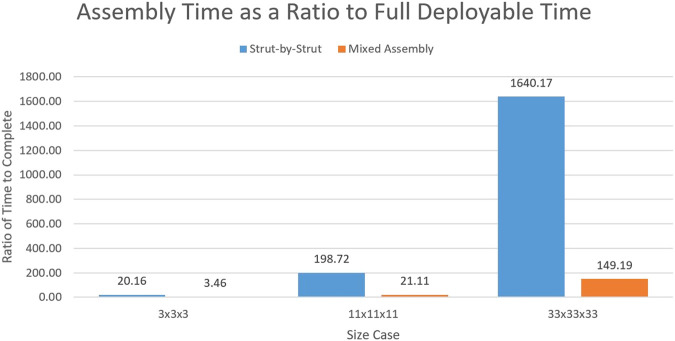
Ratio of the two assembly methods to the time of the full deployable.

The comparison using these time values can be seen in Figure 7. As the fully deployable model finished so quickly, the remaining two assembly methods showed their completion time as a ratio to the number of days to complete the full deployable unfurling. The deployable model finished in less than a day in three of the tested cases. This margin is likely to shrink when accounting for the update loop and checks are performed by the autonomous system. The time for construction for the strut-by-strut model starts out at a very reasonable value of 1.75 days total but quickly grows impractical surpassing 1500 days for the 33 × 33 × 33 case. This is due to the rate at which the components needed for this style grows as was previously shown in Figure 6. Also, finally, the mixed assembly style remains extremely achievable throughout the dimensional expansion. Here, the 33 × 33 × 33 case only increases to roughly 142 days, a stark difference from the strut-by-strut case. This proves that despite the individual tasks for the mixed assembly taking more time on average, the number of steps in the sequence is the defining influence of time in this application.

#### 3.3.3 Tight tolerance

In large structures, a prolific issue is error growth throughout the structure (Komendera et al., 2013). With so many elements coming together, small errors can compound leading to structures that quickly can fall out of acceptable error margins. Although in strut-by-strut assembly small errors can be corrected in specific cases, this can prove extremely challenging to actually implement in strut insertion style assembly. The correction of error while assembling for the strut-by-strut assembly can be more easily achievable if the truss is instead welded together at the joints. Robotic welding tasks have been able to achieve extremely tight precision (Arc Machines Inc., 2021) ^22^.

For the mixed assembly model, reducing the number of components also reduces the opportunity for this compounding error to fall outside nominal values. Mixed assembly also provides the added benefit of prefabricating the close-out-squares and deployables. This ensures that these elements in the structure can be held to tighter tolerances than may be possible with robotic space assembly.

### 3.4 Servicing

When thinking about serviceability, a key factor is how easy is it to replace a malfunctioning component. With both ISA schemes, the benefit of designing either the strut-by-strut or mixed assembly structure to be assembled by robots is inherently serviceable by robots. The same ISA steps can be reversed for disassembly, repair, or replacement, and then re-assembly. This does not hold true for the fully deployable cases, this scheme is the least serviceable because if some part is malfunctioning, the entire structure was only designed to fold for stowage and launch and deploy for operation and therefore has no pre-planning for removing a subcomponent if it is broken. Possibly the fully deployable could have some sort of emergency maintenance involving cutting an incorrect section and welding back in a replacement but that is not guaranteed depending on the type of malfunction. Strut-by-strut assembly does give most flexibility for servicing since each unit was assembled and could be replaced. Mixed assembly is also flexible in this way, just that there is the possibility of needing to replace an entire deployable if it malfunctions. Additionally, schemes with fewer parts have a slightly better chance for serviceability with fewer components to fail over the lifetime of the structure. Finally, while assembly robots are also susceptible to possible failure, the risk of this decreases with reductions in expected run-time, which could be achieved with a scheme with fewer assembly tasks.

#### 3.4.1 Reduction of single-point failure

Deployable units with higher complexity, such as the fully deployable units, are more prone to having a subcomponent fail, which can mean the failure of the entire structure. A large fully deployable structure has to complete an extremely complex series of maneuvers to result in a complete structure. In the case of the JWST, 50 major deployments consisting of 178 pin releases had to be successfully carried out (Dickinson, 2022) ^23^. If a single one failed, it could lead to the entire structure needing intervention, a challenging and sometimes impossible situation when dealing with space. The mixed assembly allows for the failure of a single deployment to be minimized to just that unit, which could be replaced with a spare unit instead of the entire structure being deemed a failure. The strut-by-strut approach is the most robust to the single-point failure since each strut is its own unit and thus spare struts could simply replace any that fail either during flight, assembly, or during the structure’s operational lifetime.

In terms of physically accessing the interior modules of the large BORG structure for servicing, either the structure could be disassembled using the reverse of the assembly process until the broken module could be accessed or the malfunctioning module could be broken and removed while still within the larger structure and a replacement brought in and installed in place. The robotic testing proposed in the next steps for the BORG demo plans to cover the problem of serviceability by showing how the robots could grab onto two adjacent nodes, remove a damaged strut, hold the nodes at the desired end positions and provide the desired forcing temporarily with a robot, and then, replace the broken strut with a correct strut and remove the robot.

## 4 Discussion

### 4.1 Weighted decision matrix

To compare the different architectures across the stages of the mission, a weighted decision matrix was created. First, key objectives from each phase of the mission were chosen for comparison. For manufacturing the number of steps and the ability to pre-integrate, advanced capabilities were considered. For stowage and transport, the number of launch vehicles required based on the volume and mass was considered. For ISA, the time taken to assemble was analyzed. Finally, for servicing, the reduction of single-point failure and serviceability was looked at. Next, a pairwise comparison chart, shown in Table 3, was created to rank each objective by comparing it to each objective and determining which had a higher priority and then adding up the sum of priority points for each objective’s row to determine its rank.

**TABLE 3:** Pairwise comparison chart- comparing each goal against all the others to rank.

Goals	Manufacturing steps	Pre-integration of advanced capabilities	LV req based on volume/mass	Time to assemble	Reduction of single point failure	Servicibility	Rank
Manufacturing steps	*-*	0	0	1	0	0	1
Pre-integration of advanced capabilities	1	-	0	1	0	0	2
LV req based on volume/mass	1	1	-	1	0	0	3
Time to assemble	0	0	0	-	0	0	0
Reduction of single point failure	1	1	1	1	-	1	5
Serviceability	1	1	1	1	0	-	4

Next, through discussion, each objective was given a weight based on its rank and how important it is when comparing the architectures. This is shown in Table 4.

**TABLE 4:** Weighted decision matrix (part 1)- giving weight to each rank.

Objective	Rank (from pairwise comparison)	Weight (determined through discussion)
Manufacturing steps	1	12%
Pre-integration of advanced capabilities	2	16%
LV req based on volume/mass	3	18%
Time to assemble	0	10%
Reduction of single-point failure	5	24%
Serviceability	4	20%
Total		100%

Then, the numbers from the previous analysis in the results section were brought into the table for each objective and compared across the different architectures for all three-sized BORG cubes. First, the raw number is recorded and then a percentage was calculated comparing the three different architectures. This is shown in Table 5.

**TABLE 5:** Weighted decision matrix (part 2)- original numbers from other sheets + percentages to compare. Cells highlighted in green indicate that architecture performed the best for the given objective, and yellow indicates when two or more architectures perform similarly well for an objective.

Objective	3 × 3 × 3 BORG			11 × 11 × 11 BORG			33 × 33 × 33 BORG		
	Strut-by-strut	Fully deployable	Mixed assembly	Strut-by-strut	Fully deployable	Mixed assembly	Strut-by-strut	Fully deployable	Mixed assembly
Manufacturing steps	1260	2025	837	45540	99825	28425	1127610	2695275	702747
	34.72%	25.44%	39.85%	36.90%	21.28%	41.82%	37.54%	20.22%	42.24%
Pre-integration of advanced capabilities	1	2	3	1	2	3	1	2	3
	16.67%	33.33%	50.00%	16.67%	33.33%	50.00%	16.67%	33.33%	50.00%
LV req based on volume/mass	1	1	1	3	0	10	60	0	233
	33.33%	33.33%	33.33%	76.92%	0.00%	23.08%	79.52%	0.00%	20.48%
Time to assemble	1.75	0.09	0.30	63.25	0.32	6.72	1566.13	0.95	142.46
	9.15%	47.90%	42.95%	5.00%	49.77%	45.22%	4.19%	49.97%	45.83%
Reduction of single-point failure	1	0	1	1	0	1	1	0	1
	50.00%	0.00%	50.00%	50.00%	0.00%	50.00%	50.00%	0.00%	50.00%
Serviceability	1	0	1	1	0	1	1	0	1
	50.00%	0.00%	50.00%	50.00%	0.00%	50.00%	50.00%	0.00%	50.00%

Finally, the final weighted decision matrix was created by multiplying the percentages from Table 5 by the overall weighting of the objectives in Table 4 to create Table 6. Then, the sum of all the categories for each architecture was found.

**TABLE 6:** Weighted decision matrix (part 3)- multiply Part 1 and 2 to get the final weighting to compare truss choices for each size. The cells highlighted in green show the architecture that cumulatively scored the highest for the weighted decision matrix for each size of the final structure.

Objective	3 × 3 × 3 BORG			11 × 11 × 11 BORG			33 × 33 × 33 BORG		
	Strut-by-strut (%)	Fully deployable (%)	Mixed assembly (%)	Strut-by-strut (%)	Fully deployable (%)	Mixed assembly (%)	Strut-by-strut (%)	Fully deployable (%)	Mixed assembly (%)
Manufacturing steps	4.17	3.05	4.78	4.43	2.55	5.02	4.51	2.43	5.07
Pre-integration of advanced capabilities	2.67	5.33	8.00	2.67	5.33	8.00	2.67	5.33	8.00
LV req based on volume/mass	6.00	6.00	6.00	13.85	0.00	4.15	14.31	0.00	3.69
Time to assemble	0.92	4.79	4.29	0.50	4.98	4.52	0.42	5.00	4.58
Reduction of single-point failure	12.00	0.00	12.00	12.00	0.00	12.00	12.00	0.00	12.00
Serviceability	10.00	0.00	10.00	10.00	0.00	10.00	10.00	0.00	10.00
TOTAL	35.75	19.18	45.08	43.44	12.86	43.69	43.91	12.76	43.34

As shown in Table 6, the fully deployable structure is always the worst option. The ISA options of strut-by-strut or mixed assembly generally score closely, with mixed assembly scoring higher for smaller-sized BORG and the difference decreasing as the size of the board increases until the strut-by-strut architecture slightly wins for the 33 × 33 × 33 case. If a specific mission had higher priority weighting for the objectives than what was chosen in Table 4, then either strut-by-strut or mixed assembly could win out in either of the larger BORG sizes since they are so close.

### 4.2 Planned verification of mixed assembly architecture

**FIGURE 8:**
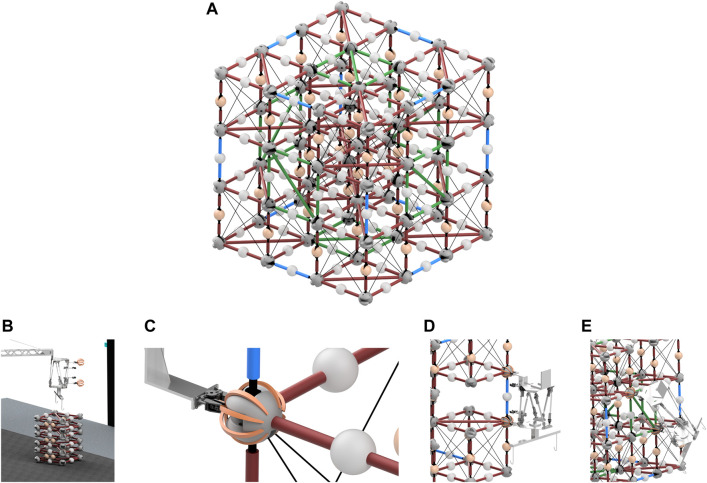
**(A)** 1.5 m BORG truss structure, **(B)** truss deployement, **(C)** grab node, **(D)** insert closeout strut, and **(E)** insert closeout panel.

To verify the mixed assembly process laid out in this paper, a series of physical assembly trials are slated to take place. This will serve as an ongoing testbed for the FASER Laboratory to continue to investigate robotic autonomy for in-space assembly and servicing applications. In order to more easily assemble the structure, the originally proposed scale of each bay having an edge length of 3 m has been scaled down to 0.5 m instead. As it is the smallest number of bays that this scheme works at, the experimental BORG will be a 3 × 3 × 3 truss resulting in an overall size of 1.5 × 1.5 × 1.5 m as shown in Figure 8. The components are constructed of 3D-printed PLA nodes, aluminum rods, steel cable, and commercial off-the-shelf internal node hardware.

#### 4.2.1 FASER Lab setup

**FIGURE 9:**
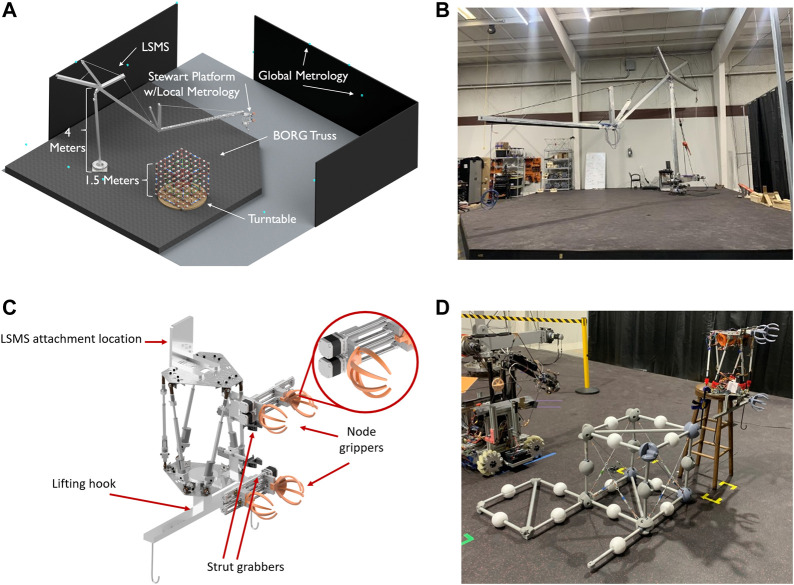
**(A)** FASER Laboratory setup for the BORG demonstration. **(B)** LSMS. **(C)** JACS. **(D)** A deployable, close-out-square, and close-out-strut test fit.

An overview of the FASER Laboratory’s demonstration workspace is shown in Figure 9. As previously mentioned, the assembly process for the mixed assembly scheme utilizes two primary robotic elements, a LRM and DM. The Lightweight Surface Manipulation System (LSMS) will serve as this long-reach manipulator and terminate in a dexterous EE called the Jigging Apparatus for Closeout Structures (JACSs). Also featured in this space is an electronic turntable to allow controllable rotation and access to all sides of the assembly and a global metrological system for object tracking and state estimation. This OptiTrak global metrological system includes 14 OptiTrack Prime^x^ 13 cameras, large black panels to block spurious reflections, and small retro-reflective markers to get accurate positions and orientations.

The LSMS is a large tendon-actuated serial arm based on NASA LaRC’s original LSMS and adapted for use at the FASER Laboratory. It stands 4 m tall above the build platform, at the shoulder joint, extends 7.5 m in length, and is capable of significant payload manipulation, up to 150 kg, albeit at coarse positioning resolution. Control is achieved by the command of joint velocities coupled with an external metrological platform. As the gross manipulator, the LSMS will be responsible for performing the task of delivering the components from the storage to the assembly area, deploying components, and securing the JACS unit throughout the duration of its tasks.

JACS is a Stewart Platform, a six degree-of-freedom dexterous manipulator, used as an end effector of LSMS. It is outfitted with in-line actuator load cells for force feedback to create compliance control during assembly. Integrated cameras and sensors allow localized metrology during assembly. On the bottom plate, it has a lifting hook to facilitate the expansion and locking of the deployment modules and transport them with the help of LSMS. A set of individually operable mobile grippers will grasp the nodal geometry to correct the misaligned nodes of the structure and compensate for deformation, essentially mimicking the force pattern of a healthy strut. While the structure is brought back to nominal conditions, the close-out elements can then be placed and secured with a set of secondary grippers, holding the structure in place during unsupported conditions. As the truss will deform during assembly stages, the JACS will be able to adjust the amount of force along the proper direction to stabilize the structure during various cases presented. Due to a few unique grasping situations required, the gripper consists of a split mobile side working in tandem with the stationary half. This mobile gripper divides into an upper and a lower section to work both in tandem and individually. Mounted to the JACS unit are cameras to be used for local metrology verification.

#### 4.2.2 Experimentation

By exploring how well this assembly scheme comes together in an experimental procedure, further insights can be gleaned to inform the BORGmixed assembly method. These assembly demonstrations will take place in the previously shown FASER Laboratory space. Due to this, all trials are subjected to Earth’s gravity. In an ideal scenario, both the robots and truss elements would be gravity offloaded to simulate a micro-g environment. Individual objects in the scene may be able to have their weight offloaded using an engine hoist. However, in a real micro-g environment, the LRM may behave with slight differences due to its lack of stiffness compared to the DM unit. This factor must be kept in mind during operations, but is currently not feasible to correct for.

Experimentation will begin with lower-level controlling of the robots with human-in-the-loop testing individual task trials such as deployment, transport, and close-out-strut insertion as shown in Figure 8. These will then transition into single layer assembly trials until a full end-to-end BORG demonstration can be performed. For this, combined teleoperation and supervised autonomy will be used to drive the gross and dexterous manipulation tasks. As confidence in the assembly procedure is gained, autonomous capabilities will be advanced with the final goal of fully autonomous assembly.

Trial list:

Each task is intended to be completed by human manipulators, driver control, and autonomous control.

• Deployment trials
• Deployment delivery
• Insertion trials
  • Close-out-strut
  • Close-out-square
• Sub-layer assembly trials
  • Layer 1 end-to-end
  • Layer 2 end-to-end
  • Layer 3 end-to-end
• Full assembly end-to-end

By completing this gambit of tests successfully, the mixed assembly scheme will be proven physically viable. By breaking up this testing procedure, each task can be verified, and if failures occur, additional measures can be explored and implemented into that specific task moving forward. These changes could be anything from a nodal redesign to a grasping location or interface change to a change in how the components are supported or many more considerations. This iteration process is critical in identifying the challenges that may arise with the physical realization of the mixed assembly scheme. These insights gained during the trials will allow for a more practical evaluation of the BORG methodology execution.

In addition, force data collected from JACS’s actuator load cells will be used to characterize stress and strain in the structure during assembly and inform efforts in reducing damage and applying targeted forces in future assembly and servicing operations. These targeted forces will allow the structure to remain stable in assembly steps where elements are unsupported by other components. By providing proper support to a damaged or incomplete assembly, stress and strain concentrations in the structure can be mitigated, deflection corrected for, and the resulting damage can be prevented. In this application, JACS is able to mimic the forces that would be naturally applied if the insertion strut was already present while it performs the insertion tasks.

When characterizing the viability of this assembly method, structural stability is paramount. The individual deployable units and the full BORG assembly will be subjected to the induced forces during the assembly process, which will be closely monitored for its own health. The deployable and completed BORG will also undergo a series of perturbation and stress tests in order to determine its stability. As these are prototype parts using non-space grade materials, these values are expected to reflect this but should still serve as a solid metric. In the case of a non-ideal outcome, steps for improving the stability, such a simulated welding or additional lockout mechanisms, will be employed.

During assembly demonstration, positional data will be collected from both the global and local metrology systems. This localized system would be given the desired final structure and build a global estimate throughout assembly from local data. The collected data from the global Optirack system will then serve as a comparison for the measurements from the JACS’s local metrology system. This will aid in both state estimation during current tasks and detection when errors are made in an assembly. This system will allow the analysis of if the joints of the modules are within desired tolerances and determine how any variance affects the overall structural geometry.

With a fully constructed 3 × 3 × 3 BORG truss, it can then serve a test stand for additional operational tasks. These can include cases of damage identification, single close-out element repairs, deployable unit repairs, cable routing, and electric continuity tests. In addition, the BORG assembly test can aid in testing out advanced autonomous algorithms for ISA and servicing.

## 5 Conclusion

This paper outlined the initial efforts of multi-robot collaborative deployment being used for the assembly of BORG truss modules, where the scale is arbitrary. This assembly scheme was evaluated alongside the two most considered methods of delivering large structures into space: fully deployable and strut-by-strut assembly, with fully deployable being the option routinely utilized. For their component construction, it was shown that due to the sheer number of struts needed and the complexity of the full deployable structure, the mixed assembly scheme was the easiest to produce. In a launch analysis, the strut-by-strut assembly and the mixed assembly schemes proved that no matter how large the size of the completed structure is, they are still able to fit in a payload fairing. However, the mixed assembly scheme used considerably more launch vehicles and was limited by the size of the individual bays. However, when approaching the question of transitioning from the stowed launch configuration to a completed structure, the mixed assembly method won out on time to complete. This is again due to the extremely high number of individual elements required to achieve large-scale strut-by-strut assembly. However, in current projections, the time to lock out a fully deployable unit still won. When approaching the lifetime a large structure in space, however, servicing damaged components is critical and this proves very challenging in a method that did not employ component-level assembly. Thus, in comparing these three methods for achieving large structures, the mixed assembly model met, if not, exceeded the viability of the strut-by-strut and fully deployable methodologies depending on the scale of the BORG structure.

## Data Availability

The raw data supporting the conclusion of this article will be made available by the authors, without undue reservation.

## Footnotes

1
https://www.nasa.gov/mission_pages/tdm/satellite-servicing.html.

2
https://www.nasa.gov/mission_pages/tdm/osam-2.html.

3
https://www.darpa.mil/program/robotic-servicing-of-geosynchronous-satellites.

4
https://www.nasa.gov/robonaut2.

5
https://www.asc-csa.gc.ca/eng/iss/canadarm2/canadarm-canadarm2-canadarm3-comparative-table.asp.

6
https://www.nasa.gov/feature/r5/.

7
https://www.darpa.mil/program/robotic-servicing-of-geosynchronous-satellites.

8
https://ntrs.nasa.gov/citations/19890004112.

9
https://webb.nasa.gov/.

10
https://exoplanets.nasa.gov/internal_resources/1254/.

11
https://www.nasa.gov/mission_pages/station/main/index.html.

12
https://www.spacex.com/media/falcon-users-guide-2021-09.pdf.

13
https://www.arianespace.com/wp-content/uploads/2011/07/Ariane5_Users-Manual_October2016.pdf.

14
https://www.ulalaunch.com/docs/default-source/rockets/delta-iv-user’s-guide.pdf.

15
https://www.ulalaunch.com/docs/default-source/rockets/atlasvusersguide2010.pdf.

16
https://explorers.larc.nasa.gov/2019APSMEX/MO/pdf_files/SLS%20mission%20planners%20guide%202018-12-19.pdf.

17
https://www.spacex.com/media/starship_users_guide_v1.pdf.

18
https://arstechnica.com/science/2022/05/rocket-report-meet-the-gravity-1-rocket-will-starship-really-cut-launch-costs/3/.

19
https://www.asc-csa.gc.ca/eng/iss/canadarm2/canadarm-canadarm2-canadarm3-comparative-table.asp.

20
http://www.collectspace.com/ubb/Forum30/HTML/000778-2.html.

21
https://www.space.com/james-webb-space-telescope-sunshield-deployment-begins.

22
https://resources.arcmachines.com/the-future-of-robotic-welding-in-manufacturing-ami/.

23
https://skyandtelescope.org/astronomy-news/deployment-james-webb-space-telescope-complete/#::text=With%20the%20successful%20deployment%20%26%20latching,178%20pins%2C%20released.
